# A Comparative Study of Phase States of the Peribacteroid Membrane from Yellow Lupin and Broad Bean Nodules

**DOI:** 10.1155/2014/527393

**Published:** 2014-04-03

**Authors:** Natalia N. Kudryavtseva, Alexis V. Sof'in, Georgiy S. Bobylev, Evgeny M. Sorokin

**Affiliations:** ^1^A.N. Bach Institute of Biochemistry of the Russian Academy of Sciences, Moscow 119071, Russia; ^2^K.A. Timiryazev Institute of Plant Physiology of the Russian Academy of Sciences, Moscow 127276, Russia

## Abstract

A comparative study of the lipid bilayer phase status and structure of the outer membrane of free-living *Bradyrhizobium* strain 359a (Nod^+^Fix^+^) and 400 (Nod^+^FixL) or *Rhizobium leguminosarum* 97 (Nod^+^Fix^+^, effective) and 87 (Nod^+^FixL, ineffective) has been carried out. Also, the effect of the symbiotic pair combination on the lipid bilayer structure of the bacteroid outer membrane and peribacteroid membrane, isolated from the nodules of *Lupinus luteus* L. or *Vicia faba* L., has been studied. As a result, it is shown that the lipid bilayer status of the bacteroid outer membrane is mainly determined by microsymbiont, but not the host plant. In the contrast, the lipid bilayer status of the peribacteroid membrane and, as a consequence, its properties depend on interaction of both symbiotic partners.

## 1. Introduction

Nitrogen-fixing symbiosis between legumes and bacteria of the* Rhizobium* genus is established through the complex interactions of the symbiotic partners [1, 2]. The lipochitin oligosaccharides, the specific signaling molecules, initiate the process of host plant nodulation. Nod factors trigger the series of responses of legume plants such as deformation of root hairs, formation of infectious threads (cytoplasmic bridge), and division of cortical cells, leading to nodules formation [3, 4]. Thus rhizobia penetrate cortical cells of the root and then into the cytoplasm of the host plant cell. The successful process development is due to the coordinated action of genes of both symbiotic partners [5–7].

Inside nodules rhizobia are modified into the endosymbiotic form of bacteroids and fix atmospheric nitrogen, providing the host plant with ammonium. In endosymbiotic conditions the survival of rhizobia is possible only in case when protective mechanisms of plant cell are suppressed, at least partially [8]. Some components of both the bacterial interface and the interface of the plant cell seem to determine whether the bacteria adopt as a symbiotic partner or the pathogenic relationships arise [9].

The process of endocytosis is characterized by a conversion from the extracellular surroundings to the intracellular one and, consequently, leads to closer interaction of* Rhizobium* cells with the surface of the plasma membrane. Now there are some data about links of the outer bacteroid membrane with regions of the peribacteroid membrane (PBM) that can serve as a proof that physical contact between the bacterial surface and the membrane of the plant cell is a factor without which it is impossible to establish normal symbiosis [10].

The presence of mutations affecting the surface of a bacterial cell may cause the symbiotically defective surface interactions. This confirms the assumption that the N_2_-fixing endosymbiosis is maintained by a physical relationship between the cell surfaces of symbiotic partners. Like a mutual recognition and interaction at early stages of symbiosis, the nitrogen-fixing activity of rhizobia inside the root nodules of the host plant is also controlled by specific contacts at later stages of nodule development [10].

A distinctive feature of endosymbiosis is existence of symbiosomes. A symbiosome is a compartment inside a host cell in which the bacteroids are surrounded by PBM, originated from the plant plasma membrane [11, 12]. PBM forms a structural and functional interface between the symbionts and plays the central role in the process of compounds exchange between the organisms. PBM formation occurs by obtaining the material from the endomembrane system [13]. This process is associated with the alterations in genome expression of both plant and bacterium; herewith specialized plant proteins are transported to the symbiosomes. Through this PBM is supplied with such components as transporter proteins and enzymes which are necessary for the symbiotic exchange processes [10].

The structure of all biological membranes is practically the same. It is a thin film of lipid and protein molecules held together by a lot of cooperative noncovalent interactions. Lipid molecules form a continuous bimolecular layer with the thickness about 50 Å [14]. State of the membrane bilayer (domain organization, the electric potential, permeability, etc.) directly affects the functioning of membrane proteins and, consequently, the majority of cellular processes, which proceed on the surface and/or inside of a biological membrane [15].

Under physiological conditions, the protein-lipid bilayer is in a liquid state preferably, but this is not unstructured liquid. It demonstrates a certain ordering of the following: there is a formation of lateral domains as a result of interaction of different types of molecules. The lateral lipid bilayer heterogeneity and the possibility to form links between the domains largely determine the functioning of the cell membranes [16].

Researches carried out so far allow saying that the lipid component of the membrane can not only affect the activity of the membrane-bound proteins and enzymes, but also completely change the cell response to certain environmental factors. Thus, it was shown that the binding force is a function of physical state of the lipid bilayer during electrostatic binding of water soluble proteins with the different charge. This force was the highest for the membranes being in the gel phase. The physical state of the membrane lipid bilayer determines also the H^+^-ATPase activity, resistance to the certain pathogens, and the temperature stress [17].

The phase state of a membrane can be estimated by the level of the fluorescence anisotropy (*r*) of a rigid fluorescent probe 1,6-diphenyl-1,3,5-hexatriene (DPH) intercalated into the hydrocarbon region of the membrane [18]. The anisotropy of the steady state fluorescence of the lipophilic probe embedded in a membrane is related to the amplitude of its rotational diffusion that depends on the structural condition of the surroundings. Thus, this carries information about viscosity, polarity, and in general about the degree of order of lipids in the hydrocarbon zone of the membrane.

The main aims of our work were comparative studies of the phase state and structure of the lipid bilayer of the outer membrane (OM) of the cells of four free-living rhizobium strains and also of the influence of the composition of symbiotic pairs on the state and structure of the outer membrane of bacteroids (bacteroid membrane, BM) and PBM, that forms the symbiosomes inside the plant cell.

## 2. Materials and Methods

To prepare specimens of PBM, OM, and plasmalemma (PL), the plants of yellow lupin* Lupinus luteus* L., cultivar “Fakel,” inoculated by rhizobium* Bradyrhizobium lupini* strains 359a (Nod^+^Fix^+^) and 400 (Nod^+^FixL), and broad bean* Vicia faba* L. inoculated by rhizobium* Rhizobium leguminosarum* strains 97 (Nod^+^Fix^+^; effective) and 87 (Nod^+^FixL; ineffective) were used. Growth and inoculation of plants, cultivation of free-living strains of the nodule-forming bacteria, and isolation of the PBM, OM, and PL specimens were performed as previously described [19].

For the fluorescence measuring, free-living bacteria, bacteroids, PL, and PBM vesicles were suspended in 0.7 M sucrose solution (pH 7.0). Then the solution of the fluorescent probe 1,6-diphenyl-1,3,5-hexatriene (DPH, Sigma, USA) in tetrahydrofuran was added to them to the final concentration of 1.5 *μ*M in the dark under constant stirring. The embedding of a fluorescent label proceeded for 40 min. Changes in the fluorescence polarization were measured by using a fluorescent spectrophotometer MPF-4 (Hitachi, Japan) at an excitation wavelength of 360 ± 5 nm and at an emission wavelength of 460 ± 10 nm. The level of the steady state fluorescence anisotropy (*r*) of a lipophilic probe embedded into the membranes was calculated as previously described [20].

Experiments were carried out at least in 3-fold repetition. The data presented in the figures are averages with standard deviation. The significant difference was defined as *P* < 0,03.

## 3. Results and Discussion

The formation of a new compartment, the symbiosome, occurs on condition that there are mutualistic interactions on the surface of bacteria and plant cells. Therefore, it was necessary to examine the changes in the lipid bilayer state of bacteria and bacteroids (microsymbiont), on the one hand, and of the PL of plant cell and PBM (macrosymbiont), on the other hand, to clarify the influence of symbiotic partners upon the state of the PBM's lipid bilayer, which determines the metabolite exchange between the symbionts and the membrane-bound proteins' activity.

In this investigation the strains forming effective (Nod^+^Fix^+^) and noneffective nodules (Nod^+^FixL) were used. The latter are morphologically normal nodules with infected cells containing endosymbiotic bacteria, but they do not testify (or testify very low) the nitrogen-fixing activity [21, 22]. In this study* B. lupini* 400 and* R. leguminosarum* 87 were such strains, Nod^+^FixL [19]. Genetic changes in the Fix^−^ mutants are detected at later stages of the symbiotic nodule development. They occur under the transformation of rhizobia in bacteroids followed by the nitrogenase induction and arising of metabolic functions required for nitrogen fixation in the plant host cells. In that regard Fix^−^ mutants differ from the Nod^−^ ones, in which the violations are observe during the early stages of the establishing of symbiosis when there is no bacterial infection, the nodule primordia formation, and, accordingly, nodule [23–26].

**Figure 1:**
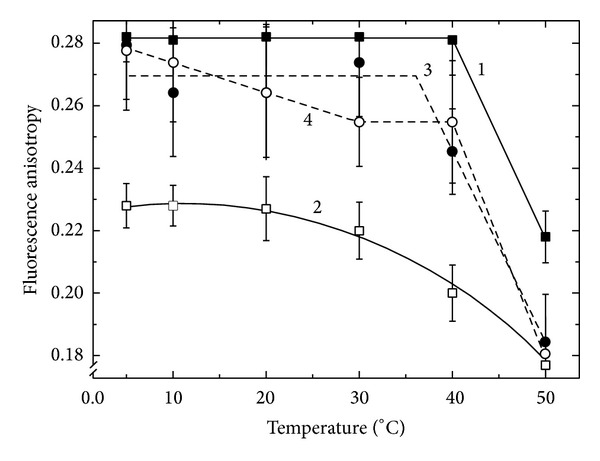
The temperature dependence of the steady state fluorescence anisotropy (*r*) of 1,6-diphenyl-1,3,5-hexatriene (DPH) embedded in a lipid matrix of the bacterial outer membrane of the free-living* B. lupini* strains 359a (1) and 400 (3) as well as isolated bacteroids formed by these strains (2 and 4, resp.).

Figure 1 shows the temperature dependence of the steady state fluorescence anisotropy of DPH in membrane preparations obtained from the free-living bacteria* B. lupini* strain 359a (Fix^+^; curve 1) and 400 (FixL; curve 3). We can see that *r* values for OM of these bacteria were initially the constants and were equal to 0,28 up to 40°C for the strain 359a and 0,27 up to 35°C for the strain 400. The fluorescence anisotropy value was reduced dramatically at higher temperatures in both cases. This was an evidence that lipid bilayer of the OM of the free-living bacteria* B. lupini* is in the solid phase, other words in the ordered state, and hence has a low permeability.

**Figure 2:**
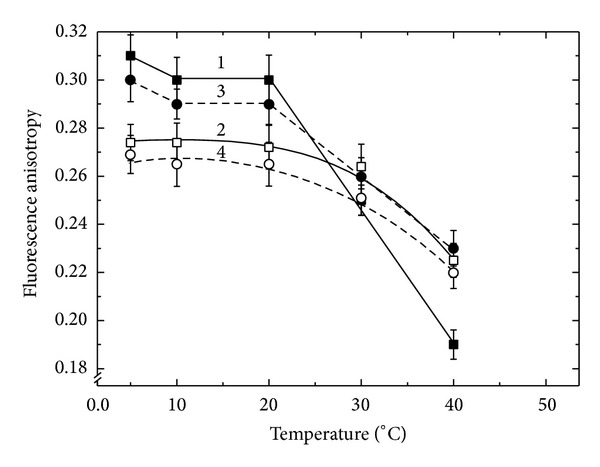
The temperature dependence of the steady state fluorescence anisotropy (*r*) of 1,6-diphenyl-1,3,5-hexatriene (DPH) embedded in a lipid matrix of the bacterial outer membrane of the free-living* R. leguminosarum* strains 97 (1) and 87 (3) as well as isolated bacteroids formed by these strains (2 and 4, resp.).

Figure 2 shows the fluorescence anisotropy data obtained for the free-living form of* R. leguminosarum*. Both strains are nitrogen fixing; however, strain 97 was effective in symbiosis with both beans and peas, whereas strain 87 formed a nitrogen fixing pair only with peas, but it was low effective in symbiosis with beans, the pair which was noncomplementary.

The dependences of the fluorescence anisotropy on temperature of the OM of these strains were characterized by very high and very close level of *r* values and, moreover, at the curves the transition phase was clearly represented which was observed at 20°C. These values of *r* indicate that the lipid bilayer of the membrane was in the solid state, in which a membrane is characterized by higher density and lower fluidity. Herewith rotation of the hydrocarbon chains around the long axis of the molecule is absent and the lateral and rotational movements of the membrane components are locked, and as a consequence the membrane permeability is sharply reduced [27, 28].

Thus, the data obtained for free-living rhizobia showed that the studied members of the families* Rhizobium* and* Bradyrhizobium* are characterized by solid-phase state of the lipid bilayer of their OM, regardless of nitrogen-fixing strains efficiency.

Transformation of the effective (Fix^+^)* B. lupini* strain into the bacteroid resulted in a marked decrease in the fluorescence anisotropy and moreover the character of the dependence *r* on the temperature changed: the curve 2 (Figure 1) had no sharp phase transition. This was an evidence of a far greater degree of fluidity (liquidity) of the bacteroid lipid bilayer in comparison with the free-living bacterium. At the same time the transformation into the bacteroid of ineffective (FixL)* B. lupini* strain (curve 4, Figure 1) did not practically lead to changes in the state of its OM.

Comparing the behavior of the fluorescence anisotropy of the abovementioned preparations one can conclude that the state of the membranes of free-living Fix^+^ and FixL strains of* B. lupini* was very close to each other, whereas the state of the membranes lipid bilayer of their bacteroids was characterized by fundamental differences. Lipid bilayer of OM of the effective strain 359a was in a solid-phase state completely ordered and as mentioned above should consequently have a low permeability. The transformation of this bacterium into the bacteroid leads to the restructuring of its membrane lipid bilayer that becomes a liquid phase by reducing the content of monounsaturated fatty acids [19]. As a result, the membrane of the bacteroid became less viscous that allowed the membrane bounded proteins to function actively [17]. The pattern was quite different for ineffective strain 400. In this strain as compared with the effective one 359a the phase transition point was shifted to lower temperatures. The OM lipid bilayer was in a solid phase, but it was loose due to the high content of iso-fatty acids [19]. The transformation of strain 400 into the bacteroid was accompanied by an increase of the degree of order in the lipid bilayer by reducing the amount of iso-fatty acids drastically [19] followed by the decrease of thickness and, hence, increasing permeability as compared with the free-living form and with bacteroids of the effective 359a strain. The study of OM of bacteroids formed by the effective (97) and ineffective (87) strains of* R. leguminosarum* showed that their lipid bilayers are in more liquid phase (with the absence of clearly expressed phase transition; Figure 2, curves 2 and 4), and as a consequence less ordered. In the case the membrane is less viscous that suggests the possibility of active functioning of membrane bound proteins. The changes observed are similar to those for the bacteroids of effective strain* B. lupini* 359a. At the same time the difference between the bacteroids from the effective and ineffective nodules formed by noncomplementary strain 87 was almost absent. This is probably due to the fact that the strain 87 is effective and only under these experiment's conditions reveals itself to be ineffective because of the noncomplementarity to the host plant.

**Figure 3:**
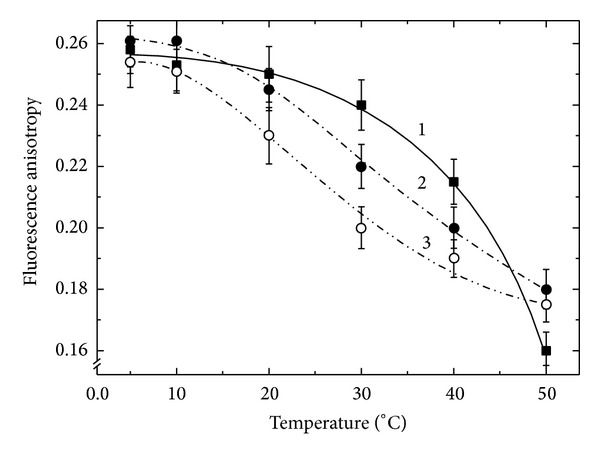
The temperature dependence of the steady state fluorescence anisotropy (*r*) of 1,6-diphenyl-1,3,5-hexatriene (DPH) embedded in a lipid matrix of the plasmalemma specimens from* Lupinus luteus* L. (1) as well as the peribacteroid membrane specimens isolated from the lupin nodules, formed by the* B. lupini* strains 359a (2) and 400 (3).

Figure 3 illustrates the dependence of the anisotropy of the steady state fluorescence on the temperature for PL of lupin (curve 1) compared to the PBM of Fix^+^ (curve 2) and FixL (curve 3) of the lupin nodules.

It can be pointed out that there was no explicit phase transition that was a common feature of these membranes. Furthermore, there were also some differences between the PL and the newly formed symbiotic membranes. While the dependence of the fluorescence anisotropy on temperature for PL was a convex curve, in the case of PBM the one was *S*-shaped. This indicated that the lipid matrix of the PBM specimens had similar properties and differed from that of the preparations of PL.

The data obtained show that at the temperature range from 0 to 20°C only 20–30% of the PBM bilayer of the lupin nodules was in a liquid state. At higher temperatures the shapes of the curves remained practically identical and the *r* values were similar. This indicates that at the temperature above 20°C the share of liquid fraction in the PBM was increased by 60–70%. On this basis one can argue that the disorder degree of the lipid bilayer in both specimens of PBM from the lupin nodules was close. Hence it can be assumed that the diffusion value both for its own components and for ones penetrating through it must be close. In other words it is believed that the permeabilities of the two membranes from lupin nodules should be close.

It is interesting to note that the data presented for PBM from the lupin nodules are very similar to that obtained for isolated membranes of the chloroplast, another compartment of a plant cell [20]. The similarity of the probing curves of the PBM and the chloroplast membrane may indicate that for the symbiosome the main thing is its own dominating feature that is the physical separation of bacteroide, as an extrinsic body, from the nodule plant cell cytoplasm.

**Figure 4:**
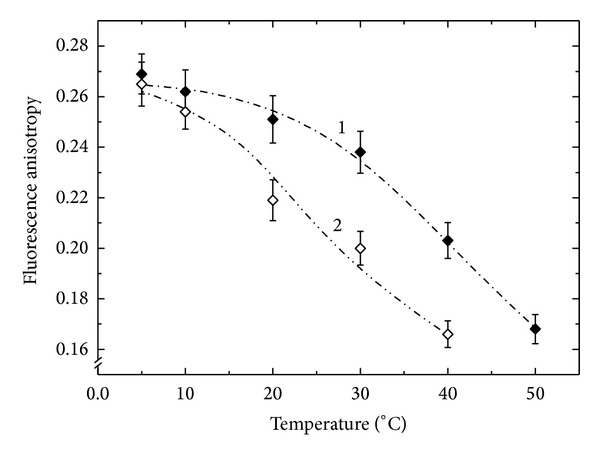
The temperature dependence of the steady state fluorescence anisotropy (*r*) of 1,6-diphenyl-1,3,5-hexatriene (DPH) embedded in a lipid matrix of the peribacteroid membrane specimens isolated from the broad bean* Vicia faba* L. nodules, formed by the* R. leguminosarum* strains 97 (1) and 87 (2).

Comparison of the dependences of the anisotropy of the steady state fluorescence on the temperature for the specimens from the nodules of* Vicia faba* (Figure 4) shows that only in the symbiosis with bacterium that is noncomplimentary to the plant (strain 87) a membrane was formed with fully destructurized lipid bilayer (Figure 4, curve 2). In the case of an effective pair the run of a curve of the dependence of the fluorescence anisotropy on the temperature resembled to that for the PBM from lupin under the condition of the effective symbiosis with the strain 359a.

The above data imply that the conditions of the lipid matrix of the PBM specimens from effective nodules of different host plants are very similar (Figures 3 and 4). The distinctions in the fatty acids composition of the PBM of different origin that we detected earlier [19] have obviously no effect on the properties of their lipid matrix. It can be assumed that these distinctions are probably not substantial for the lipid bilayer condition and, consequently, for the membrane in general. This indicates the existence of dynamic changes in the lipids conformation and reflects probably the general trend that is observed in the formation of intracellular compartments of the plant cell.

The latter conclusion, however, cannot be attributed to the PBM isolated from nodules formed by noncomplimentary strain 87 with* Vicia faba* plants (Figure 4, curve 2, see below).

The comparison of the composition and content of fatty acids in the PL and PBM showed that the permeability that was determined by the level of unsaturated fatty acids should be higher in the case of PBM compared to PL [29]. On the other hand, the high content of monounsaturated fatty acids in the PBM from ineffective nodules determines a high disorder degree of the membrane lipid bilayer. This probably causes lower temperature of the phase transition and, in accordance with lower unsaturation index [19], of the reduced membrane dynamics [17], that is, the physiological membrane fluidity, and as a result decreased enzymatic activity of its membrane bound proteins [30].

Such conclusion can be confirmed by the data obtained during a study of ATPase activity. It was shown that a reduction of the Na^+^, K^+^-ATPase activity correlates with an increase of *r* value of the fluorescence anisotropy [30]. Even more, this reduction seems to be due to changes in the enzyme molecule conformation in consequence of changes in the structure of the membrane lipid bilayer surrounding ATPase.

So, based on the data obtained, it was possible to speak about the identity of the conditions of the PBM lipid bilayer in case of effective symbioses (Figures 3 and 4) and their difference with the PBM from the noncomplimentary pair of* V. faba-R. leguminosarum* strain 87 (Figure 4, curve 2) that had a disordered and fully permeable membrane. Considering the PBM lipid bilayer condition as a parameter reflecting the efficiency of the symbiosis, one can conclude that a symbiotic pair that is formed by a noncomplementary strain supports a point of view that a symbiosis is a special case of parasitism. In the case, the host is trying to protect themselves from the parasite resulting in observed premature aging and rapid destruction of the cytoplasm of infected cells and consequently the impossibility of existence of a nonnitrogen-fixing symbiont (parasite) [31].

## 4. Conclusion

Thus, on the basis of our data, it can be argued that, irrespectively of the efficiency of nitrogen-fixing symbiosis, the condition and properties of the BM lipid bilayer are mainly determined by the strain of microsymbiont and do not depend on the host plant. At the same time the differences in the condition and, therefore, in the properties of PBM depend on the interaction of both symbiosis partners and obviously affect the activity of its membrane bound proteins. Since the membrane lipid matrix is not a barrier for O_2_ [17], the PBM function is the formation of the compartment intended primarily for physical separation of bacteroids, but not to limit the flow of oxygen for them which, as it is well known, dramatically affect the activity of the bacteroid nitrogenase. So, the flow of oxygen that is available to bacteroids is only limited by the amount, distribution, and state of the leghemoglobin in the cytoplasm of the infected host plant cells [32].
